# Task Type Affects Location of Language-Positive Cortical Regions by Repetitive Navigated Transcranial Magnetic Stimulation Mapping

**DOI:** 10.1371/journal.pone.0125298

**Published:** 2015-04-30

**Authors:** Theresa Hauck, Noriko Tanigawa, Monika Probst, Afra Wohlschlaeger, Sebastian Ille, Nico Sollmann, Stefanie Maurer, Claus Zimmer, Florian Ringel, Bernhard Meyer, Sandro M. Krieg

**Affiliations:** 1 Department of Neurosurgery, Klinikum rechts der Isar, Technische Universität München, Munich, Germany; 2 Faculty of Linguistics, Philology, and Phonetics, University of Oxford, Oxford, United Kingdom; 3 Section of Neuroradiology, Department of Radiology, Klinikum rechts der Isar, Technische Universität München, Munich, Germany; University Of Cambridge, UNITED KINGDOM

## Abstract

**Objectives:**

Recent repetitive TMS (rTMS) mapping protocols for language mapping revealed deficits of this method, mainly in posterior brain regions. Therefore this study analyzed the impact of different language tasks on the localization of language-positive brain regions and compared their effectiveness, especially with regard to posterior brain regions.

**Methods:**

Nineteen healthy, right-handed subjects performed object naming, pseudoword reading, verb generation, and action naming during rTMS language mapping of the left hemisphere. Synchronically, 5 Hz/10 pulses were applied with a 0 ms delay

**Results:**

The object naming task evoked the highest error rate (14%), followed by verb generation (13%) and action naming (11%). The latter revealed more errors in posterior than in anterior areas. Pseudoword reading barely generated errors, except for phonological paraphasias.

**Conclusions:**

In general, among the evaluated language tasks, object naming is the most discriminative task to detect language-positive regions via rTMS. However, other tasks might be used for more specific questions.

## Introduction

Neuroscientists use various noninvasive methods to examine human cortical language function, especially functional MRI (fMRI), magnetoencephalography (MEG) [1], and electroencephalography (EEG). The first studies evaluating repetitive transcranial magnetic stimulation (TMS) for this purpose were performed in the early 1990s [2]. At that time, the investigators examined a wide range of stimulation frequencies and tasks (object naming, counting, word reading) [3,4]. It was shown that TMS is able to evoke language disruption during different language tasks, although the very limited spatial resolution did not allow analysis of different patterns of the identified language-positive brain regions for each task.

With the development of navigated TMS (nTMS), TMS gains increasing importance for neurosurgical but also general neuroscientific language mapping [5]. It was already shown that repetitive nTMS (rTMS) is able to identify anterior language eloquent areas quite accurately [6,7]. Yet Picht et al. showed that rTMS combined with an object naming task revealed a high sensitivity and also a relatively high negative predictive value (NPV) compared to intraoperative direct cortical stimulation (DCS) but still there was a considerable number of false negative language areas. Thus, with our present knowledge of rTMS, positive responses are not reliable enough for clinical use, and neurosurgeons have to rely on outlining language *negative* brain regions [7]. We therefore have to further improve the NPV in order to provide an even more reliable negative mapping by rTMS. However, we then have to avoid false negative results, which especially appeared in posterior language regions—i.e., angular (anG) and supramarginal gyrus (SMG)—in a previous trial [7].

Thus, the rationale of this study was to improve rTMS language mapping of posterior language areas. The authors therefore wanted to ascertain, if other language tasks than object naming were more specific to posterior language regions. Hence, we tested four different language tasks during rTMS and examined the impact of these language tasks on reliability and distribution of language-positive regions. Principally, the tasks should be easy feasible and therefore be suitable for usage during awake craniotomies. Furthermore, we selected tasks which are commonly used in the language production assessment and which are supposed to involve posterior language regions. So additionally to the object naming task standardly used in pre- and intraoperative language mapping, we tested a pseudoword reading task, for which is described a wide distribution of left-hemispheric activation clusters [8] Due to the large number of reported cases of noun-specific and verb-specific aphasic patients and the supposed distinct neural network representation of nouns and verbs [9,10], we furthermore included an action naming task and a verb generation task.

At least to our knowledge, this is the first lesion-based study comparing different naming and reading tasks in healthy volunteers during rTMS.

## Materials and Methods

### Study subjects

Twenty right-handed volunteers (ten female, ten male) without any neurological disorders were enrolled. The mean age was 24.6 ± 1.7 (range 22–29; Table 1). All subjects were German native speakers and had no additional mother tongue. Right-handedness was proven by the Edinburgh handedness test [11].

**Table 1:** Age, pain, motor threshold (MT) and baseline performance.

age		24.6 ± 1.7	-
pain (VAS)	convexity	1.7 ± 1.6	p < 0.0001
	temporal	5.4 ± 2.2	
motor threshold intensity (% stimulator output)		33.1 ± 4.8	-
representative correct baseline pictures (out of a dataset of 100)	Object naming	90.9 ± 4.5	p <0.0001
	Pseudoword reading	95.6 ± 2.4	
	Verb generation	88.2 ± 4.9	
	Action naming	87.4 ± 5.1	
statistical dependence between error rate during baseline performing and error rate induced by stimulation: Spearman’s rank correlation coefficient (r_s_)	Object naming	0.0253	-
	Pseudoword reading	0.4733	
	Verb generation	-0.1169	
	Action naming	0.1140	

Inclusion criteria were an age above 18 years and written informed consent. Exclusion criteria were previous seizures, bilateral or left handedness, second mother tongue, pathological findings on cranial MRI, aberrant medical history, developmental language deficits, any neurological impairment, and general TMS exclusion criteria (e.g., pacemaker, deep brain stimulation, or cochlear implant) [12].

Age of the volunteers, pain score according to the visual analogue scale (VAS), RMT (= resting motor threshold; % stimulator output), and representative correct baseline pictures presented as mean ± standard deviation (SD); Spearman’s rank correlation coefficient r_s_ for testing statistical dependence between error rate during baseline performing and error rate induced by stimulation

### Study design

The subjects underwent language mapping of the left hemisphere with four different language tasks to examine their impact on error rate and language location.

### Ethics

The local ethical committee of the Technische Universität München approved the experimental procedures (registration number: 2793/10) in accordance with the Declaration of Helsinki. Written informed consent was obtained from all volunteers prior to navigational MRI.

### Navigational MRI scan

All participants received a navigational MRI for neuronavigation prior to rTMS language mapping. It was performed on a 3 Tesla MR scanner (Achieva 3T, Philips Medical Systems, The Netherlands B.V.) by use of an 8-channel phased array head coil. For anatomical co-registration, a 3D fast field echo sequence (TR/TE 8.3/3.9 ms, 1mm^3^ isovoxel covering the whole head, 5 min and 56 s acquisition time) without intravenous contrast administration was used. Subsequently, the 3D dataset was transferred to the rTMS system via DICOM standard.

### rTMS language mapping

#### Experimental setup

All subjects underwent rTMS language mapping of the left hemisphere using the Nexstim eXimia NBS system version 4.3 and a NexSpeech module (Nexstim Oy, Helsinki, Finland).

The first author performed all mapping sessions. Each language mapping followed the same procedure as published earlier, yet with another picture to trigger an interval of 0 instead of 300 ms [7,13]. In short, prior to each session, the Resting Motor Threshold (RMT) was determined by motor mapping of the cortical representation of the left-sided hand area (right-sided abductor pollicis brevis muscle and abductor digiti minimi muscle) as described previously [14]. Because the RMT serves as a measure for cortex excitability, the stimulus intensity was adjusted to the RMT [15] and was 100% RMT in all mapping sessions. Ten bursts were applied via each rTMS train with 5 Hz over 2 s.

#### Language tasks

We used four different language tasks: object naming, pseudoword reading, verb generation, and action naming. Each of the tasks consisted of a set of 100 items and was performed in German.

The object naming task consisted of colored pictures of common objects similar to those listed in the established Snodgrass and Vanderwart picture set (1980) [6,7]. The shown objects had to be named without article.

For the pseudoword reading task, the subjects had to read aloud 50 pseudowords randomly mixed with 50 real words that served as a control. We used items of a German word list by Felty et al. [16]. Felty et al. generated a disyllabic word list containing nouns, verbs, and adjectives in the CVCCVC structure (C = Consonant, V = Vowel). The pseudowords were derived from those words.

During the verb generation task, the subjects had to build verbs out of common objects demonstrated visually by pictures on the screen.

The action naming task contained pictures that showed daily activities (e.g., sleeping, dancing), so the instruction was to name these actions without using a noun.

Within one task, items were randomly displayed on a screen 60 cm in front of the subject.

#### Language mapping procedure

The pictures/words had to be named or read quickly and precisely. Before starting with the actual stimulation during one task, the subjects had to perform baseline testing for that task. All misnamed or misread items were discarded from the stimulus sequence (they were not shown during stimulation); correctly named items were counted and documented (Table 1). Thus, we considered and recorded inter-individual differences in the vocabulary and during stimulation, the subjects only had to form words that he or she perfectly recognized without stimulation. Pictures of the object naming, action naming, and verb generation task were displayed for 700 ms. Because some of the pseudowords were rather long, we used a display time of 1.0 s for this task. The inter picture interval (IPI) of all four tasks was 3.0 s. Magnetic pulses were applied simultaneously with the item presentation. Based on Indefrey et al.’s proposal of time course of human language processing [17], the authors reduced the PTI from 300 ms in earlier studies [e.g. [18];[19]] to 0 ms. In general, one language mapping session required 90–120 min per subject. Pain in temporal areas and the remaining hemisphere (convexity) was documented separately with the visual analogue scale (VAS): 0 = no pain, 10 = maximum pain (Table 1).

Video recording of baseline performance as well as of the actual language mapping session was conducted for objective and detailed post-hoc language analysis [15].

#### Stimulated points

**Fig 1:**
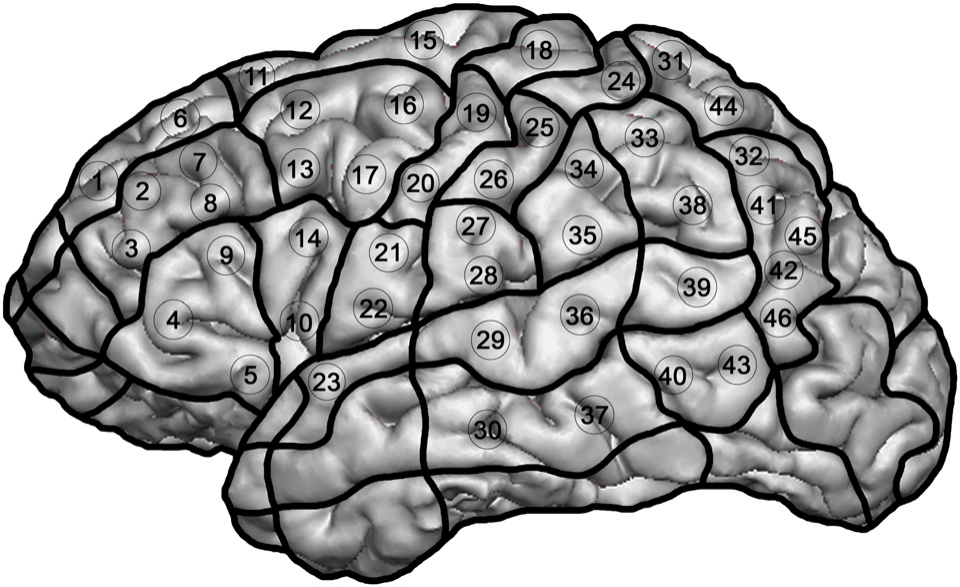
Outline of the 46 stimulated cortical spots. Each point was stimulated for three times.

We stimulated 46 previously determined cortical spots, which were distributed over the left hemisphere and easy to reproduce in the cortical 3D reconstruction of healthy subjects (Fig 1). Prior to each mapping session, those 46 spots were tagged on the 3D MRI. The extent of stimulated cortical areas had to be restricted due to unacceptable pain. Hence, we did not apply rTMS to the orbital part of the inferior frontal gyrus (orIFG), polar superior and polar middle temporal gyrus (polSTG, polMTG), anterior middle temporal gyrus (aMTG) and polar superior (polSFG), or polar middle (polMFG) and polar inferior frontal gyrus (polIFG). Furthermore, since stimulation intensity decreased below 50 V/m (increasing distance between skin and brain), the inferior temporal gyrus (ITG) was also not mapped [19]. Within one language task, each of the 46 spots was stimulated three times, which equals 138 stimulations per task. The coil was placed tangential to the skull in a strictly anterior-posterior field orientation [3,4,15]. At the cortical region of interest, the accepted minimum field strength was 55 V/m and ranged from 55–80 V/m across participants.

For appropriate data description, the localization of language-positive points was described according to the cortical parcellation system (CPS, Table 2), which was presented by Corina et al. [20].

**Table 2:** Abbrevations of the cortical parcellation system (CPS).

Abbreviation	Anatomy
anG	angular gyrus
aSMG	anterior supramarginal gyrus
aSTG	anterior superior temporal gyrus
dPoG	dorsal post-central gyrus
dPrG	dorsal pre-central gyrus
mMFG	middle middle frontal gyrus
mMTG	middle middle temporal gyrus
mPoG	middle post-central gyrus
mPrG	middle pre-central gyrus
mSFG	middle superior frontal gyrus
mSTG	middle superior temporal gyrus
opIFG	opercular inferior frontal gyrus
orIFG	orbital part of the inferior frontal gyrus
pMFG	posterior middle frontal gyrus
pMTG	posterior middle temporal gyrus
polIFG	polar inferior frontal gyrus
polMFG	polar middle frontal gyrus
polMTG	polar middle temporal gyrus
polSFG	polar superior frontal gyrus
polSTG	polar superior temporal gyrus
pSFG	posterior superior frontal gyrus
pSMG	posterior supramarginal gyrus
pSTG	posterior superior temporal gyrus
SPL	superior parietal lobe
trIFG	triangular inferior frontal gyrus
vPoG	ventral post-central gyrus
vPrG	ventral pre-central gyrus

Anatomical names and abbreviations of the cortical parcellation system (CPS) according to Corina et al. (2005).

Furthermore, we formed two groups of stimulated cortical spots: the anterior group contained stimulation points 1 to 22; the posterior group points 23 to 46.

#### Data analysis

The analysis of the recorded videos of all mapping sessions was performed as described in earlier publications [5,7,15], blinded to stimulated cortical spots as well as to subject and previous results.

Directly after baseline analysis, task performance during stimulation was evaluated. Any language impairment was directly compared to the baseline. Evoked errors were divided into different error categories: No response errors, performance errors, hesitations, neologisms, semantic paraphasias, and phonological paraphasias—as outlined in previous studies [18,21]—and nominalization. The latter category is only relevant to the verb generation and action naming tasks, when subjects did not succeed in finding the appropriate verb, but the noun. All error types were summarized in the error category “all errors.” If at least one out of the three stimulations per spot caused any error compared to the baseline, the spot was considered as language positive. Errors attributed to muscle stimulation or discomfort were discarded from further analysis. For comparison of tasks, we calculated error rates. The error rate resulted from the number of elicited language errors per number of stimulations and is expressed as a percentage.

### Statistics

Representative correct baseline pictures, pain (VAS), and RMT were presented as mean ± standard deviation (SD). For testing differences between the numbers of correctly named baseline pictures in each language task, Friedman’s test for nonparametric matched groups was performed. Furthermore, we tested correlation between the baseline error rate of each task and the error rate during stimulation of that task. Therefore we performed nonparametric Spearman correlation with a two-tailed p value and 95% confidence interval. Differences among distribution of error rates per stimulation point in different tasks were tested using Friedman’s test followed by Dunn’s post hoc test. A value of p<0.05 was considered significant (GraphPad Prism 6.0, La Jolla, CA, USA).

## Results

### Stimulation-related incidents and discomfort

For the first time in our mapping experience, one out of the twenty included subjects developed vegetative symptoms including perspiration and nausea, and complained about intensive discomfort during RMT determination prior to rTMS so that we stopped the investigation of this participant. The remaining 19 participants tolerated the stimulation well.

### Errors in different language tasks

**Fig 2:**
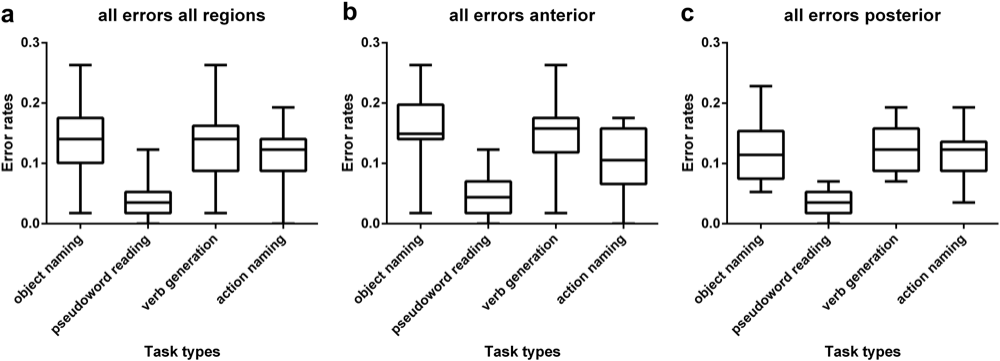
Error rates per stimulation spot during object naming, pseudoword reading, verb generation, and action naming. Results are presented for all regions (a), for anterior regions (b), and for posterior regions (c).

First, when generally comparing the overall error rates, we observed the highest error rate in the object naming task, followed by the verb generation and action naming tasks (Table 3). Pseudoword reading barely generated errors (Tables 3–7, Fig 2a).

**Table 3:** Summary of naming errors.

Error category	Object naming			Pseudoword reading			Verb generation			Action naming			Friedman's test		
	all	ant.	post.	all	ant.	post.	all	ant.	post.	all	ant.	post.	all	ant.	post.
No response	1.6	1.9	1.4	0	0.1	0	1.6	1.4	1.8	1.8	1.1	2.4	p < 0.0001	p = 0.0020	p < 0.0001
Hesitations	9.9	10.8	9.1	1.5	1.8	1.2	9.5	10.3	8.7	7.8	8	7.6	p < 0.0001	p < 0.0001	p < 0.0001
Performance	1.3	1.4	1.2	0.8	1	0.7	1	1.1	0.8	0.6	0.6	0.7	p = 0.1084	p = 0.2309	p = 0.3384
Phonological	0.2	0.2	0.3	1.6	1.8	1.5	0.4	0.7	0.1	0.4	0.7	0.1	p < 0.0001	p = 0.0011	p = 0.0004
Semantic	0.6	1	0.2	0	0	0	0.3	0.4	0.3	0.3	0.3	0.2	p = 0.0041	p = 0.0066	p = 0.3916
Neologism	0	0.1	0	0	0	0.1	0.1	0.1	0.1	0	0.1	0			
Nominalization	0	0	0	0	0	0	0.6	0.6	0.6	0.2	0.1	0.2			
All errors	13.7	15.3	12.1	4	4.6	3.4	13.5	14.5	12.5	11.1	10.8	11.3	p < 0.0001	p < 0.0001	p < 0.0001

Summary of naming errors in [%] induced by rTMS: error ratios of each error type in the four tested language tasks. Differences among distribution of error rates per stimulation point in different tasks were tested using Friedman’s test.

**Table 4:** Naming errors during object naming.

	No response		Performance		Hesitation		Neologism		Phonological		Semantic		Totals	
Stimulation point	Errors	Rate	Errors	Rate	Errors	Rate	Errors	Rate	Errors	Rate	Errors	Rate	Errors	Rate
1	0	0.00	0	0.00	1	0.02	0	0.00	0	0.00	0	0.00	1	0.02
2	0	0.00	0	0.00	3	0.05	0	0.00	0	0.00	0	0.00	3	0.05
3	1	0.02	0	0.00	1	0.02	0	0.00	0	0.00	1	0.02	3	0.05
4	4	0.07	0	0.00	4	0.07	0	0.00	0	0.00	0	0.00	8	0.14
5	0	0.00	0	0.00	8	0.14	0	0.00	0	0.00	0	0.00	8	0.14
6	2	0.04	0	0.00	5	0.09	0	0.00	0	0.00	2	0.04	9	0.16
7	0	0.00	0	0.00	5	0.09	0	0.00	0	0.00	1	0.02	6	0.11
8	2	0.04	1	0.02	8	0.14	0	0.00	0	0.00	0	0.00	11	0.19
9	3	0.05	4	0.07	6	0.11	0	0.00	0	0.00	2	0.04	15	0.26
10	2	0.04	0	0.00	5	0.09	0	0.00	0	0.00	1	0.02	8	0.14
11	0	0.00	0	0.00	12	0.21	0	0.00	0	0.00	0	0.00	12	0.21
12	0	0.00	1	0.02	4	0.07	0	0.00	0	0.00	3	0.05	8	0.14
13	0	0.00	0	0.00	7	0.12	0	0.00	0	0.00	1	0.02	8	0.14
14	0	0.00	2	0.04	10	0.18	0	0.00	0	0.00	0	0.00	12	0.21
15	2	0.04	2	0.04	4	0.07	1	0.02	0	0.00	1	0.02	10	0.18
16	2	0.04	1	0.02	9	0.16	0	0.00	0	0.00	0	0.00	12	0.21
17	1	0.02	1	0.02	7	0.12	0	0.00	1	0.02	0	0.00	10	0.18
18	0	0.00	0	0.00	8	0.14	0	0.00	0	0.00	0	0.00	8	0.14
19	2	0.04	0	0.00	6	0.11	0	0.00	1	0.02	0	0.00	9	0.16
20	0	0.00	3	0.05	6	0.11	0	0.00	0	0.00	0	0.00	9	0.16
21	3	0.05	1	0.02	10	0.18	0	0.00	0	0.00	0	0.00	14	0.25
22	0	0.00	1	0.02	6	0.11	0	0.00	0	0.00	1	0.02	8	0.14
23	2	0.04	2	0.04	6	0.11	0	0.00	0	0.00	1	0.02	11	0.19
24	0	0.00	0	0.00	10	0.18	0	0.00	0	0.00	0	0.00	10	0.18
25	0	0.00	0	0.00	6	0.11	0	0.00	1	0.02	0	0.00	7	0.12
26	0	0.00	0	0.00	3	0.05	0	0.00	0	0.00	0	0.00	3	0.05
27	1	0.02	1	0.02	9	0.16	0	0.00	0	0.00	1	0.02	12	0.21
28	2	0.04	0	0.00	11	0.19	0	0.00	0	0.00	0	0.00	13	0.23
29	0	0.00	2	0.04	2	0.04	0	0.00	0	0.00	0	0.00	4	0.07
30	1	0.02	1	0.02	11	0.19	0	0.00	0	0.00	0	0.00	13	0.23
31	1	0.02	1	0.02	7	0.12	0	0.00	0	0.00	0	0.00	9	0.16
32	0	0.00	2	0.04	1	0.02	0	0.00	0	0.00	0	0.00	3	0.05
33	0	0.00	0	0.00	6	0.11	0	0.00	0	0.00	0	0.00	6	0.11
34	0	0.00	2	0.04	4	0.07	0	0.00	0	0.00	0	0.00	6	0.11
35	0	0.00	1	0.02	6	0.11	0	0.00	0	0.00	0	0.00	7	0.12
36	2	0.04	0	0.00	4	0.07	0	0.00	1	0.02	1	0.02	8	0.14
37	3	0.05	0	0.00	0	0.00	0	0.00	0	0.00	0	0.00	3	0.05
38	1	0.02	1	0.02	4	0.07	0	0.00	0	0.00	0	0.00	6	0.11
39	0	0.00	0	0.00	4	0.07	0	0.00	1	0.02	0	0.00	5	0.09
40	1	0.02	1	0.02	6	0.11	0	0.00	0	0.00	0	0.00	8	0.14
41	0	0.00	0	0.00	3	0.05	0	0.00	0	0.00	0	0.00	3	0.05
42	0	0.00	0	0.00	5	0.09	0	0.00	0	0.00	0	0.00	5	0.09
43	1	0.02	0	0.00	3	0.05	0	0.00	0	0.00	0	0.00	4	0.07
44	1	0.02	0	0.00	5	0.09	0	0.00	0	0.00	0	0.00	6	0.11
45	1	0.02	1	0.02	4	0.07	0	0.00	1	0.02	0	0.00	7	0.12
46	2	0.04	1	0.02	4	0.07	0	0.00	0	0.00	0	0.00	7	0.12
SUM	43	0.02	33	0.01	259	0.10	1	0.00	6	0.00	16	0.01	358	0.14
ANTERIOR	24	0.02	17	0.01	135	0.11	1	0.00	2	0.00	13	0.01	192	0.15
POSTERIOR	19	0.01	16	0.01	124	0.09	0	0.00	4	0.00	3	0.00	166	0.12

Summary of all naming errors induced by rTMS trains during object naming. Results are demonstrated as absolute values and error rates per stimulation point, as sum of all stimulation points, and separately for anterior and posterior regions.

**Table 5:** Naming errors during pseudoword reading.

	No response		Performance		Hesitation		Neologism		Phonological		Totals	
Stimulation point	Errors	Rate	Errors	Rate	Errors	Rate	Errors	Rate	Errors	Rate	Errors	Rate
1	0	0.00	0	0.00	0	0.00	0	0.00	0	0.00	0	0.00
2	0	0.00	0	0.00	1	0.02	0	0.00	0	0.00	1	0.02
3	0	0.00	0	0.00	1	0.02	0	0.00	1	0.02	2	0.04
4	0	0.00	0	0.00	0	0.00	0	0.00	0	0.00	0	0.00
5	0	0.00	0	0.00	0	0.00	0	0.00	2	0.04	2	0.04
6	0	0.00	0	0.00	2	0.04	0	0.00	1	0.02	3	0.05
7	0	0.00	0	0.00	3	0.05	0	0.00	2	0.04	5	0.09
8	0	0.00	0	0.00	2	0.04	0	0.00	1	0.02	3	0.05
9	0	0.00	1	0.02	3	0.05	0	0.00	1	0.02	5	0.09
10	0	0.00	4	0.07	1	0.02	0	0.00	2	0.04	7	0.12
11	0	0.00	0	0.00	2	0.04	0	0.00	1	0.02	3	0.05
12	0	0.00	0	0.00	0	0.00	0	0.00	1	0.02	1	0.02
13	0	0.00	1	0.02	0	0.00	0	0.00	0	0.00	1	0.02
14	0	0.00	2	0.04	1	0.02	0	0.00	1	0.02	4	0.07
15	0	0.00	0	0.00	0	0.00	0	0.00	2	0.04	2	0.04
16	0	0.00	0	0.00	0	0.00	0	0.00	0	0.00	0	0.00
17	0	0.00	2	0.04	0	0.00	0	0.00	2	0.04	4	0.07
18	0	0.00	0	0.00	3	0.05	0	0.00	0	0.00	3	0.05
19	1	0.02	0	0.00	3	0.05	0	0.00	0	0.00	4	0.07
20	0	0.00	3	0.05	0	0.00	0	0.00	2	0.04	5	0.09
21	0	0.00	0	0.00	0	0.00	0	0.00	2	0.04	2	0.04
22	0	0.00	0	0.00	0	0.00	0	0.00	1	0.02	1	0.02
23	0	0.00	0	0.00	1	0.02	0	0.00	2	0.04	3	0.05
24	0	0.00	1	0.02	0	0.00	0	0.00	2	0.04	3	0.05
25	0	0.00	0	0.00	1	0.02	0	0.00	0	0	1	0.02
26	0	0.00	2	0.04	0	0.00	0	0.00	0	0.00	2	0.04
27	0	0.00	1	0.02	1	0.02	1	0.02	0	0.00	3	0.05
28	0	0.00	0	0.00	0	0.00	0	0.00	2	0.04	2	0.04
29	0	0.00	0	0.00	0	0.00	0	0.00	0	0.00	0	0.00
30	0	0.00	1	0.02	2	0.04	0	0.00	0	0.00	3	0.05
31	0	0.00	0	0.00	2	0.04	0	0.00	2	0.04	4	0.07
32	0	0.00	0	0.00	1	0.02	0	0.00	0	0.00	1	0.02
33	0	0.00	0	0.00	1	0.02	0	0.00	2	0.04	3	0.05
34	0	0.00	0	0.00	2	0.04	0	0.00	0	0.00	2	0.04
35	0	0.00	0	0.00	1	0.02	0	0.00	1	0.02	2	0.04
36	0	0.00	0	0.00	0	0.00	0	0.00	0	0.00	0	0.00
37	0	0.00	2	0.04	0	0.00	0	0.00	1	0.02	3	0.05
38	0	0.00	0	0.00	0	0.00	0	0.00	1	0.02	1	0.02
39	0	0.00	2	0.04	0	0.00	0	0.00	2	0.04	4	0.07
40	0	0.00	0	0.00	0	0.00	0	0.00	2	0.04	2	0.04
41	0	0.00	0	0.00	1	0.02	0	0.00	0	0.00	1	0.02
42	0	0.00	0	0.00	0	0.00	0	0.00	0	0.00	0	0.00
43	0	0.00	0	0.00	1	0.02	0	0.00	2	0.04	3	0.05
44	0	0.00	0	0.00	0	0.00	0	0.00	0	0.00	0	0.00
45	0	0.00	0	0.00	2	0.04	0	0.00	0	0.00	2	0.04
46	0	0.00	0	0.00	1	0.02	0	0.00	1	0.02	2	0.04
SUM	1	0.00	22	0.01	39	0.01	1	0.00	42	0.02	105	0.04
ANTERIOR	1	0.00	13	0.01	22	0.02	0	0.00	22	0.02	58	0.05
POSTERIOR	0	0.00	9	0.01	17	0.01	1	0.00	20	0.01	47	0.03

Summary of all naming errors induced by rTMS trains during pseudoword reading. Results are demonstrated as absolute values and error rates per stimulation point, as sum of all stimulation points, and separately for anterior and posterior regions.

**Table 6:** Naming errors during verb generation.

	No response		Performance		Hesitation		Neologism		Phonological		Semantic		Nominalization		Totals	
Stimulation point	Errors	Rate	Errors	Rate	Errors	Rate	Errors	Rate	Errors	Rate	Errors	Rate	Errors	Rate	Errors	Rate
1	0	0.00	0	0.00	1	0.02	0	0.00	0	0.00	0	0.00	0	0.00	1	0.02
2	1	0.02	0	0.00	2	0.04	0	0.00	0	0.00	0	0.00	0	0.00	3	0.05
3	0	0.00	1	0.02	9	0.16	0	0.00	0	0.00	0	0.00	0	0.00	10	0.18
4	1	0.02	0	0.00	3	0.05	0	0.00	1	0.02	0	0.00	0	0.00	5	0.09
5	0	0.00	1	0.02	6	0.11	0	0.00	0	0.00	0	0.00	0	0.00	7	0.12
6	0	0.00	1	0.02	7	0.12	0	0.00	0	0.00	0	0.00	0	0.00	8	0.14
7	2	0.04	0	0.00	1	0.02	0	0.00	0	0.00	0	0.00	1	0.02	4	0.07
8	1	0.02	0	0.00	5	0.09	0	0.00	2	0.04	0	0.00	0	0.00	8	0.14
9	0	0.00	1	0.02	7	0.12	0	0.00	0	0.00	1	0.02	0	0.00	9	0.16
10	0	0.00	1	0.02	7	0.12	0	0.00	2	0.04	0	0.00	0	0.00	10	0.18
11	3	0.05	0	0.00	5	0.09	0	0.00	0	0.00	0	0.00	1	0.02	9	0.16
12	2	0.04	0	0.00	4	0.07	0	0.00	0	0.00	0	0.00	0	0.00	6	0.11
13	0	0.00	0	0.00	10	0.18	0	0.00	1	0.02	1	0.02	0	0.00	12	0.21
14	0	0.00	0	0.00	5	0.09	0	0.00	2	0.04	0	0.00	1	0.02	8	0.14
15	0	0.00	3	0.05	6	0.11	0	0.00	0	0.00	1	0.02	0	0.00	10	0.18
16	1	0.02	0	0.00	8	0.14	0	0.00	0	0.00	0	0.00	0	0.00	9	0.16
17	1	0.02	2	0.04	7	0.12	1	0.02	0	0.00	1	0.02	3	0.05	15	0.26
18	1	0.02	0	0.00	7	0.12	0	0.00	0	0.00	1	0.02	1	0.02	10	0.18
19	1	0.02	1	0.02	8	0.14	0	0.00	0	0.00	0	0.00	0	0.00	10	0.18
20	2	0.04	0	0.00	8	0.14	0	0.00	0	0.00	0	0.00	0	0.00	10	0.18
21	0	0.00	1	0.02	5	0.09	0	0.00	1	0.02	0	0.00	0	0.00	7	0.12
22	1	0.02	2	0.04	8	0.14	0	0.00	0	0.00	0	0.00	0	0.00	11	0.19
23	0	0.00	2	0.04	4	0.07	0	0.00	0	0.00	0	0.00	1	0.02	7	0.12
24	1	0.02	1	0.02	6	0.11	0	0.00	0	0.00	0	0.00	0	0.00	8	0.14
25	1	0.02	0	0.00	5	0.09	0	0.00	0	0.00	0	0.00	2	0.04	8	0.14
26	1	0.02	1	0.02	9	0.16	0	0.00	0	0.00	0	0.00	0	0.00	11	0.19
27	2	0.04	0	0.00	7	0.12	0	0.00	0	0.00	0	0.00	0	0.00	9	0.16
28	2	0.04	0	0.00	5	0.09	0	0.00	0	0.00	1	0.02	1	0.02	9	0.16
29	1	0.02	1	0.02	3	0.05	0	0.00	0	0.00	0	0.00	0	0.00	5	0.09
30	4	0.07	0	0.00	5	0.09	0	0.00	0	0.00	0	0.00	0	0.00	9	0.16
31	1	0.02	1	0.02	2	0.04	0	0.00	1	0.02	0	0.00	0	0.00	5	0.09
32	0	0.00	1	0.02	3	0.05	0	0.00	0	0.00	0	0.00	0	0.00	4	0.07
33	1	0.02	0	0.00	6	0.11	1	0.02	0	0.00	0	0.00	0	0.00	8	0.14
34	1	0.02	0	0.00	3	0.05	0	0.00	0	0.00	0	0.00	0	0.00	4	0.07
35	1	0.02	1	0.02	5	0.09	0	0.00	0	0.00	0	0.00	0	0.00	7	0.12
36	0	0.00	0	0.00	6	0.11	0	0.00	0	0.00	0	0.00	0	0.00	6	0.11
37	2	0.04	0	0.00	5	0.09	0	0.00	1	0.02	2	0.04	1	0.02	11	0.19
38	0	0.00	1	0.02	4	0.07	0	0.00	0	0.00	0	0.00	0	0.00	5	0.09
39	2	0.04	0	0.00	2	0.04	1	0.02	0	0.00	0	0.00	0	0.00	5	0.09
40	0	0.00	0	0.00	6	0.11	0	0.00	0	0.00	0	0.00	1	0.02	7	0.12
41	0	0.00	0	0.00	5	0.09	0	0.00	0	0.00	0	0.00	0	0.00	5	0.09
42	1	0.02	1	0.02	4	0.07	0	0.00	0	0.00	0	0.00	1	0.02	7	0.12
43	0	0.00	0	0.00	8	0.14	0	0.00	0	0.00	0	0.00	1	0.02	9	0.16
44	2	0.04	0	0.00	5	0.09	0	0.00	0	0.00	1	0.02	0	0.00	8	0.14
45	2	0.04	0	0.00	3	0.05	0	0.00	0	0.00	0	0.00	0	0.00	5	0.09
46	0	0.00	1	0.02	8	0.14	0	0.00	0	0.00	0	0.00	0	0.00	9	0.16
SUM	42	0.02	25	0.01	248	0.09	3	0.00	11	0.00	9	0.00	15	0.01	353	0.13
ANTERIOR	17	0.01	14	0.01	129	0.10	1	0.00	9	0.01	5	0.00	7	0.01	182	0.15
POSTERIOR	25	0.02	11	0.01	119	0.09	2	0.00	2	0.00	4	0.00	8	0.01	171	0.13

Summary of all naming errors induced by rTMS trains during verb generation. Results are demonstrated as absolute values and error rates per stimulation point, as sum of all stimulation points, and separately for anterior and posterior regions.

**Table 7:** Naming errors during action naming.

	No response		Performance		Hesitation		Neologism		Phonological		Semantic		Nominalization		Totals	
Stimulation point	Errors	Rate	Errors	Rate	Errors	Rate	Errors	Rate	Errors	Rate	Errors	Rate	Errors	Rate	Errors	Rate
1	0	0.00	0	0.00	0	0.00	0	0.00	0	0.00	0	0.00	0	0.00	0	0.00
2	0	0.00	0	0.00	1	0.02	0	0.00	0	0.00	1	0.02	0	0.00	2	0.04
3	0	0.00	0	0.00	3	0.05	0	0.00	0	0.00	0	0.00	0	0.00	3	0.05
4	1	0.02	1	0.02	4	0.07	0	0.00	1	0.02	0	0.00	0	0.00	6	0.11
5	0	0.00	0	0.00	6	0.11	0	0.00	0	0.00	0	0.00	0	0.00	6	0.11
6	0	0.00	0	0.00	5	0.09	0	0.00	0	0.00	0	0.00	0	0.00	5	0.09
7	0	0.00	1	0.02	5	0.09	0	0.00	0	0.00	0	0.00	0	0.00	8	0.14
8	0	0.00	0	0.00	5	0.09	0	0.00	2	0.04	0	0.00	0	0.00	6	0.11
9	0	0.00	0	0.00	4	0.07	0	0.00	0	0.00	0	0.00	0	0.00	4	0.07
10	1	0.02	1	0.02	5	0.09	0	0.00	2	0.04	0	0.00	0	0.00	7	0.12
11	1	0.02	0	0.00	6	0.11	0	0.00	0	0.00	0	0.00	0	0.00	7	0.12
12	2	0.04	0	0.00	4	0.07	0	0.00	0	0.00	1	0.02	0	0.00	9	0.16
13	1	0.02	0	0.00	2	0.04	0	0.00	1	0.02	0	0.00	0	0.00	3	0.05
14	0	0.00	2	0.04	5	0.09	0	0.00	2	0.04	0	0.00	0	0.00	10	0.18
15	1	0.02	2	0.04	6	0.11	0	0.00	0	0.00	0	0.00	0	0.00	9	0.16
16	3	0.05	0	0.00	3	0.05	0	0.00	0	0.00	0	0.00	0	0.00	6	0.11
17	1	0.02	0	0.00	8	0.14	0	0.00	0	0.00	0	0.00	1	0.02	10	0.18
18	0	0.00	0	0.00	5	0.09	0	0.00	0	0.00	1	0.02	0	0.00	6	0.11
19	0	0.00	0	0.00	7	0.12	0	0.00	0	0.00	1	0.02	0	0.00	9	0.16
20	1	0.02	0	0.00	6	0.11	1	0.02	0	0.00	0	0.00	0	0.00	8	0.14
21	1	0.02	0	0.00	8	0.14	0	0.00	1	0.02	0	0.00	0	0.00	9	0.16
22	1	0.02	0	0.00	2	0.04	0	0.00	0	0.00	0	0.00	0	0.00	3	0.05
23	0	0.00	0	0.00	6	0.11	0	0.00	0	0.00	0	0.00	0	0.00	6	0.11
24	3	0.05	0	0.00	3	0.05	0	0.00	0	0.00	1	0.02	0	0.00	7	0.12
25	0	0.00	1	0.02	6	0.11	0	0.00	0	0.00	0	0.00	0	0.00	7	0.12
26	2	0.04	1	0.02	3	0.05	0	0.00	0	0.00	0	0.00	1	0.02	7	0.12
27	3	0.05	1	0.02	2	0.04	0	0.00	0	0.00	0	0.00	1	0.02	7	0.12
28	1	0.02	0	0.00	7	0.12	0	0.00	0	0.00	0	0.00	0	0.00	8	0.14
29	0	0.00	0	0.00	7	0.12	0	0.00	0	0.00	1	0.02	0	0.00	8	0.14
30	3	0.05	0	0.00	6	0.11	0	0.00	0	0.00	0	0.00	0	0.00	9	0.16
31	1	0.02	0	0.00	4	0.07	0	0.00	1	0.02	0	0.00	0	0.00	5	0.09
32	2	0.04	2	0.04	3	0.05	0	0.00	0	0.00	0	0.00	0	0.00	7	0.12
33	0	0.00	0	0.00	7	0.12	0	0.00	0	0.00	0	0.00	0	0.00	7	0.12
34	0	0.00	0	0.00	3	0.05	0	0.00	0	0.00	0	0.00	0	0.00	3	0.05
35	3	0.05	1	0.02	4	0.07	0	0.00	0	0.00	1	0.02	0	0.00	9	0.16
36	3	0.05	1	0.02	6	0.11	0	0.00	0	0.00	0	0.00	1	0.02	11	0.19
37	1	0.02	0	0.00	1	0.02	0	0.00	1	0.02	0	0.00	0	0.00	2	0.04
38	1	0.02	1	0.02	3	0.05	0	0.00	0	0.00	0	0.00	0	0.00	5	0.09
39	0	0.00	0	0.00	3	0.05	0	0.00	0	0.00	0	0.00	0	0.00	3	0.05
40	1	0.02	0	0.00	6	0.11	0	0.00	0	0.00	0	0.00	0	0.00	7	0.12
41	2	0.04	0	0.00	3	0.05	0	0.00	0	0.00	0	0.00	0	0.00	5	0.09
42	1	0.02	0	0.00	5	0.09	0	0.00	0	0.00	0	0.00	0	0.00	6	0.11
43	0	0.00	0	0.00	5	0.09	0	0.00	0	0.00	0	0.00	0	0.00	5	0.09
44	2	0.04	1	0.02	4	0.07	0	0.00	0	0.00	0	0.00	0	0.00	7	0.12
45	3	0.05	0	0.00	1	0.02	0	0.00	0	0.00	0	0.00	0	0.00	5	0.09
46	1	0.02	0	0.00	6	0.11	0	0.00	0	0.00	0	0.00	0	0.00	8	0.14
SUM	47	0.02	16	0.01	204	0.08	1	0.00	11	0.00	7	0.00	4	0.00	290	0.11
ANTERIOR	14	0.01	7	0.01	100	0.08	1	0.00	9	0.01	4	0.00	1	0.00	136	0.11
POSTERIOR	33	0.02	9	0.01	104	0.08	0	0.00	2	0.00	3	0.00	3	0.00	154	0.11

Summary of all naming errors induced by rTMS trains during action naming. Results are demonstrated as absolute values and error rates per stimulation point, as sum of all stimulation points, and separately for anterior and posterior regions.

#### Object naming

**Fig 3:**
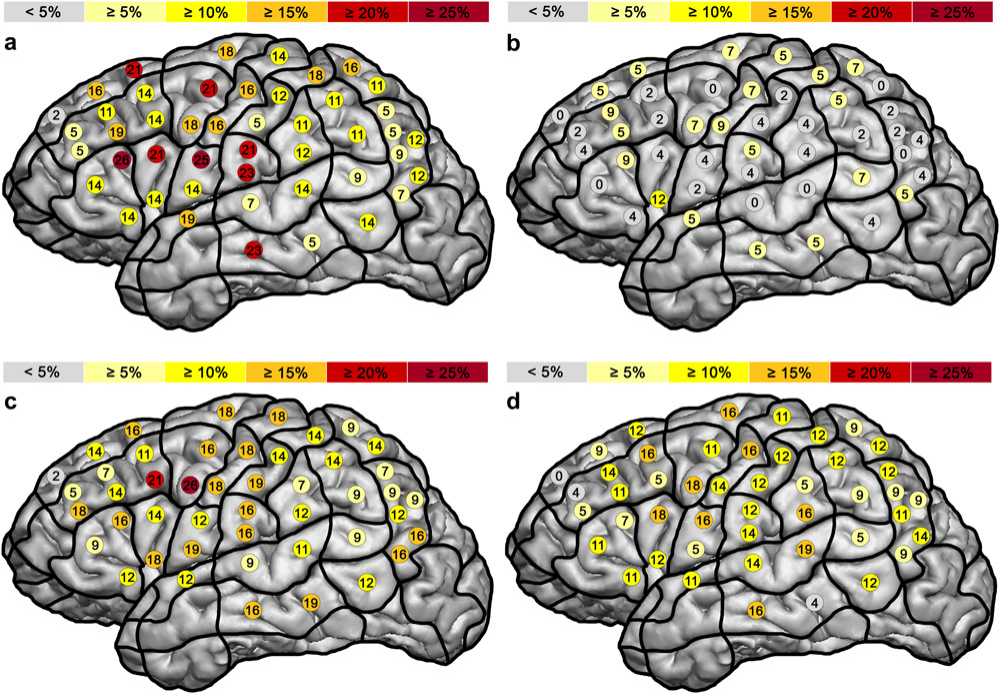
Overall error rates revealed by language mapping via rTMS. Distribution of elicited naming errors while performing object naming (a), pseudoword reading (b), verb generation (c), and action naming (d).

At each of the 46 stimulated spots, we observed language disruption during stimulation. When performing the object naming task, triangular inferior frontal gyrus (trIFG), ventral pre-central gyrus (vPrG), ventral post-central gyrus (vPoG), and middle middle temporal gyrus (mMTG) included points with the highest error rates (Fig 3a). In anterior areas, more errors were elicited than in posterior areas (Tables 3 and 4, Fig 2b and 2c).

**Fig 4:**
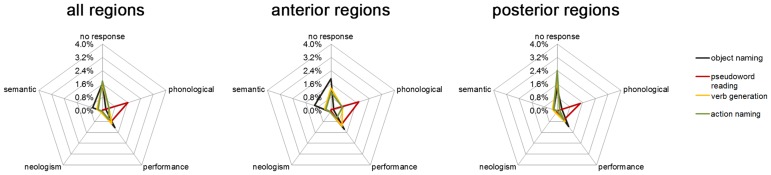
Error type (%) differences across task types. Error rates per error category during object naming, pseudoword reading, verb generation, and action naming shown for all regions, anterior regions, and posterior regions.

No response errors were mainly located in trIFG, vPrG, or mMTG and generally occurred more recently in anterior than in posterior regions (Tables 3 and 4). Compared to the other language tasks, it elicited the highest no response error rate in anterior brain regions (Fig 4).

The most frequently occurring error type, “hesitation”, was elicited in 9.9% of all stimulations. During this task, we observed the highest number of performance errors (Tables 3 and 4, Fig 4). Phonologic errors, semantic errors, and neologisms were very rare (error rate ≤0.6%; Tables 3 and 4, Fig 4). However, no other language task elicited more semantic errors (Fig 4).

#### Pseudoword reading

Concerning the sum of all errors, the highest error rate was elicited during stimulation of the opercular inferior frontal gyrus (opIFG; Fig 3b). The pseudoword reading task failed to elicit a considerable number of no response errors, performance errors, and neologisms (error rates ≤0.8%; Tables 3 and 5, Fig 4). Also hesitation errors appeared comparatively rarely (Tables 3 and 5). Nevertheless, with regard to the other tested language tasks, pseudoword reading clearly revealed the highest number of phonological errors (Tables 3 and 5, Fig 4). Errors of this category were widely distributed over the hemisphere. Phonological errors were elicited more frequently in anterior regions than in posterior regions (Tables 3 and 5, Fig 4).

#### Verb generation

Again, we observed language disruption at each of the 46 stimulated points (Fig 3c). The overall error rate in anterior regions was higher than in posterior regions (Tables 3 and 6). CPS regions where language impairment could be observed most frequently were middle middle frontal gyrus (mMFG) and posterior middle frontal gyrus (pMFG; Fig 3c). Contrary to the object naming task, there were fewer no response errors in anterior than in posterior regions during the verb generation task (Tables 3 and 6, Fig 4). Moreover, it was not possible to elicit more of the rare semantic, neologism, and phonological errors with this task (error rate ≤0.4%; Tables 3 and 6, Fig 4). We observed 15 nominalization errors (Table 6). This error type showed the highest error rate (5.3%) in pMFG.

#### Action naming

The action naming task provided language disruption in all stimulated sites, except for stimulation point number 1 (Fig 3d). There were more errors in posterior regions than in anterior regions (Tables 3 and 7). CPS regions containing the points with highest error rates were mSTG, opIFG, and pMFG during the first investigation (Fig 3d). No response errors were distributed in posterior regions (Tables 3 and 7, Fig 4). Hesitations as well as performance errors, semantic errors, and neologisms occurred less frequently than they did during object naming and verb generation (Tables 3 and 7, Fig 4). Phonologic errors appeared more often than they did during the object naming task and tended to be localized mainly in anterior regions (Tables 3 and 7). Stimulation elicited few nominalization errors (we counted four of this type; Table 7).

### Task comparison

Differences among the number of correctly named baseline pictures were statistically significant (p < 0.0001; Table 1), which is indicative that some of the used tasks were easier to perform than others. Generally, the pseudoword reading task seemed to be less demanding than the naming and generation tasks.

The low Spearman’s rank correlation coefficients show that there is no correlation between error rates in baseline testing and error rates induces by rTMS stimulation (Table 1). There was also a statistically significant difference among the distribution of all errors during pseudoword reading and the other used tasks showing that each task was discriminative concerning the involved functional pattern (p<0.0001; Table 3; Fig 3). Furthermore, in anterior regions, object naming differed significantly from action naming (Fig 4). With regard to no response errors, hesitations, and phonological errors, we also observed significant differences between pseudoword reading and the remaining tasks in all regions (p<0.0001; Table 3; Fig 3) as well as in anterior (no response: p = 0.002; hesitations: p<0.0001; phonological errors: p = 0.001; Table 3) and posterior regions (p<0.0001). The occurrence of semantic errors revealed differences between tasks regarding all regions (p = 0.004) and anterior regions (p = 0.007), but not posterior regions (p = 0.392). Considering performance errors, there seemed to be no differences among the four language tasks (all regions: p = 0.108; anterior: p = 0.231; posterior: p = 0.338; Table 3).

## Discussion

For neurosurgeons as well as neuroscientists, it is crucial to have a reliable armamentarium of techniques for noninvasively localizing cortical language function. On most modalities, the object naming task was shown to provide sufficient results due to involving numerous cortical and subcortical sub-functions. Furthermore, its superiority to e.g. counting was recently demonstrated [22]. In recent as well as in past TMS studies, it is widely distributed; until now, within our four different tested language tasks, as far as to our knowledge object naming has been the only visual task used in rTMS investigations combined with a navigated system (e.g., [7,15,19]). As mentioned above, limitations of this technique seem to concern posterior perisylvian brain regions especially [7]. Other language tasks involving semantics might be more suitable for posterior brain regions. In the following, we discuss our results of the tested language tasks in relation to current models of language production and compare them with respect to their applicability to preoperative planning via rTMS.

### Object naming

The underlying cortical regions in word processing during object naming were, simply put, pMTG for semantic and phonological representations, opIFG, where the phonological information is sent for motor language processing (syllabification), and vPrG to initiate a motor command for articulation [17].

We found higher error rates in anterior (especially trIFG, opIFG, vPrG, and mMTG) regions, which corresponds to previous findings of false negative points during object naming when comparing rTMS to DCS (Fig 3, Tables 3 and 4). It is likely that errors in anterior regions—especially no response errors—are caused by disrupting articulatory planning in opIFG and speech-motor commands in vPrG and therefore rather represent a disruption of automatic speech processing than language production. Furthermore, we obtained a considerably high error rate in the middle and posterior superior frontal gyrus (mSFG, pSFG; Fig 3, Table 4). SFG does not appear in any of the established language processing models. Moreover, the role of pSFG as a key component in working memory [23] and the role of mSFG in facilitating semantic comprehension, when a word’s meaning depends on semantic context [24] were discussed. Because stimulation in this area mainly evoked hesitation errors, the SFG might play a supporting role in language processing during object naming. Posterior cortical regions involved in word processing reported by previous studies were particularly the inferior parietal gyrus, STG and MTG [17,25–27]. Contrary to MTG, we did not achieve a strikingly increased error rate in SMG, anG, and STG compared to error rates in anterior regions by use of the object naming task (Fig 3, Table 4).

### Pseudoword reading

Previous studies of the functional organization of reading words and pseudowords revealed—in greatly simplified terms—left-hemispheric activation in visual brain areas, in the anterior fusiform, angular and middle temporal gyrus for lexical and semantic processing, in the inferior parietal cortex in general for spelling-sound conversion, and in the inferior frontal gyrus for phonological output [8].

One of the cognitive models of reading, Coltheart’s DRC model, describes a lexical route for known and irregular words in which the reader has access to orthographic and phonological lexica and the semantic system. The nonlexical route is for reading pseudowords and contains a rule system for converting graphemes into phonemes [28]. Phonological dyslexia is assumed to be associated with impairments in this route—i.e., difficulties in pseudoword reading—so that pseudoword reading tasks are widely used in this context (e.g., [29,30]). Hence, as expected, when testing pseudoword reading during stimulation, we primarily evoked phonological errors.

Comparing our results with the above-named brain regions involved in reading mechanisms, we cannot make any statements about the fusiform gyrus: as mentioned above, we did not apply stimulation trains in this area because of the increasing distance between skin and brain and the consequently insufficient stimulation intensity.

Regarding error rates of all error types in stimulated areas, we observe a higher error rate in the ventro-lateral angular gyrus (vAG; Fig 3), which corresponds to earlier reports of involvement of the anG in reading [31,32]. Phonological store was shown to be localized in pSTG [33,34]. According to this finding, our investigations revealed a high error rate in pSTG (Fig 3, Table 5). Against expectation, our data did not show a considerable high occurrence of errors in inferior parietal gyrus. The highest error rates were found in the dorsal Broca’s area (Fig 3, Table 5). The reason for this high error rate could be that both the nonlexical and lexical route—pseudoword and word reading—revert to the inferior frontal gyrus for conversion of the information into articulatory codes [35].

### Verb generation

Verb generation can be used to test functions of thematic association and selection for action [36]. Since the instruction was to build verbs out of visually presented objects, the underlying cortical regions coincide to a large extent with the above-named regions involved in word processing during object naming. Generation of the semantically appropriate verb to the concrete noun (what can the object do or what can be done with the object, respectively) was found to be associated with the IFG, especially in trIFG and opIFG [37,38]. In our investigation, errors also appeared frequently in these areas (Fig 3, Table 6). Semantically driven word retrieval in verb generation is furthermore related to the MFG [38]. In this region, or more exactly in pMFG, we obtained the highest error rate during this task (Fig 3, Table 6). Because 32% of the participants were unable to pronounce a verb during the verb generation task and instead named the presented object, we implemented another type of error: nominalization. Errors of this category also appeared mainly in pMFG, which is indicative that this region might possibly be essential to the production of verbs as general language production was still possible. Previous studies demonstrated activation of the anterior cingulate cortex during verb generation due to competition among alternative responses [39]. Furthermore, several studies described the involvement of the cerebellum, especially the dentate nucleus, in verb generation tasks [40,41]. The fact that these parts of the brain cannot be investigated via rTMS is a great disadvantage compared to other imaging methods like fMRI or positron emission tomography (PET).

### Action naming

The large number of reported cases of noun-specific and verb-specific aphasic patients in the last few decades suggests a possible double dissociation and has led to the claim that words that are nouns and words that are verbs are represented in distinct neural networks with noun processing in temporal areas and verb processing in frontal areas [9,42]. Yet other authors also suggested that a common neural system processes nouns and verbs [43] and that different findings of localization relies on different linguistic and/or general processing demands [44]. During awake surgery, selective impairment in object and action naming has been confirmed [10]. Therefore, we decided to conduct an action naming task in addition to the object naming task. Admittedly, with our current action naming task design, we were not able to strip off semantic associations between the lexical classes of nouns and verbs. Nevertheless, the aim of this study was not to reason out the neurobiological basis of noun and verb processing, but to compare different easily feasible language tasks concerning their potential to better detect language-positive sites in posterior brain regions via rTMS. Indeed, although we found language impairment in anterior and posterior regions using this task, when regarding all errors and no response errors, we obtained the highest error rates in posterior regions among the four tasks (Fig 4, Tables 3 and 7). Because the occurrence of no response errors during stimulation suggests a crucial role of the corresponding inhibited site in single-word production, no response errors are of great significance. Assuming that semantic factors play an important role in differences in the localization of nouns and verbs [45], our data indicates that semantic distinctions are predominantly associated with posterior brain regions, especially with mSTG, mMTG, and perisylvian aSMG (Fig 3).

### Task comparison

Existing literature has shown large-scaled distribution of object naming function within the putative cortical language areas [46–48]. This also corresponds to our findings regarding the number of overall errors and the frequency of different elicited error types: The object naming task caused the highest overall, semantic, and performance error rates and a similar amount of no response errors and neologisms within the four language tests (Fig 3, Tables 3–7). Thus, it appears to be the most discriminative task. Nevertheless, we should keep in mind that the applied error categories have been adjusted to an object naming task and therefore embrace most of the potentially occurring errors. This fact could further contribute to the low number of errors evoked by the pseudoword reading task. In addition, reading constitutes an automatized mechanism, whereas in naming and generation tasks the subject has to choose between several alternatives. The lower percentage of recognized baseline pictures in naming tasks than in reading indicates that the former are more challenging and thus might involve more brain regions simultaneously. Furthermore, by analyzing error rates during baseline and stimulation, we found no correlation between the number of errors in baseline performance and during stimulation. This indicates that a subject with a low number of recognized baseline pictures does not have to show many errors during stimulation, so errors during stimulation seem to be independent from baseline performance. Regarding pseudoword reading, this task does not seem to be as suitable for clinical utility as the visual naming tasks. Nevertheless, rTMS in combination with a pseudoword reading task offers a feasible technique for neuropsychological research.

Comparing overall errors, pseudoword reading differs significantly from all other tasks (Table 3), which also implies the distinct underlying cortical mechanisms of reading and naming or generation. Furthermore, we obtained significant differences between the object and action naming tasks in anterior regions. This indicates that distinct cortical regions are involved in the processing of verbs and nouns.

Regarding the distribution of different error types, we obtained comparable error rates of no response errors during object naming, verb generation, and action naming. We demonstrated that rTMS is actually able to identify different spatial patterns of involved brain areas for each task (Figs 3 and 4).

In our study, the verb generation task revealed no distinct advantages over the other language tests: Stimulation during object naming generally caused more errors, and errors in posterior regions appeared more frequently in action naming than during verb generation.

### Clinical implications

Positive naming sites during object naming are considered essential to language, and there is evidence that their resection causes language deficits after neurosurgical procedures. Nevertheless, even when preserving areas during resection, in which cortical stimulation causes disruption of the naming task, patients may exhibit postoperative language decline or alexia [22,49]. This indicates that the performance of only one task may be insufficient as language impairment might only be provoked when stimulating the cortical regions essential to that task. Ideally then, patients should be mapped by using several tasks. Because the concentration of volunteers or patients declined during the rTMS mapping over time, we have to choose a small number of different tasks that best suit each patient to maximize the effectiveness of our mapping. As a lesion-based method highly comparable to DCS, rTMS provides a reasonable approach not only for preoperative planning but also to detect the most suitable tasks prior to surgery for each individual patient.

### Limitations

One of the general limitations of rTMS is that stimulation cannot be applied in all brain regions due to pain. In this study, one participant’s investigation even had to be stopped. Moreover, important cortical language structures—e.g., the fusiform gyrus for reading—cannot be examined. Discomfort is also the reason why we should apply only short trains of stimulation. Furthermore, timed correlation of stimulus presentation and stimulation is evident. Therefore, presentation of sentences during stimulation is unsuitable when using this rTMS protocol. Yet the processing of phrases is necessary to investigate grammatical classes [50] and to distinguish between speech and language impairment when eliciting hesitations, performance, or no response errors. This also outlines restrictions of the current protocol.

As another limitation of this study, we did not measure naming, reading, or generation latencies. Consequently, accessing time course of word production in different language tasks was not possible. Moreover, hesitation errors—the most frequently occurring error type—were only compared to baseline testing, so a certain degree of subjectivity cannot be excluded.

Lastly, this study does not contain information about involvement of white matter fibers. Because previous studies proposed a hodotopical model of language organization [47,51–53], virtual lesion studies at cortical as well as subcortical levels are indicated. Therefore, rTMS combined with diffusion tensor imaging (DTI) seems to be a useful technique for future language analysis studies.

## Conclusion

To summarize, this study provides rTMS data of four language tasks and their impact on error rates and location of language-positive regions. Pseudoword reading revealed a low overall error rate. However, the location of positive sites is mostly consistent with the current models of reading processes and evoked the highest number of phonological errors. In our study, the verb generation task showed no distinct advantages over the other language tests. Action naming revealed the highest error rate in posterior regions and therefore seems to be applicable when aiming to map the posterior perisylvian region. However, in general, the object naming task is the most discriminative test to detect language-positive regions in rTMS, at least out of the four tests examined.

This study shows the easy application of different language tasks via rTMS. According to the subject’s condition and problem, a corresponding test can be chosen to improve preoperative language mapping.
